# New IMB16-4 Nanoparticles Improved Oral Bioavailability and Enhanced Anti-Hepatic Fibrosis on Rats

**DOI:** 10.3390/ph15010085

**Published:** 2022-01-11

**Authors:** Xia Niu, Xiaomei Wang, Bingyu Niu, Yucheng Wang, Hongwei He, Guiling Li

**Affiliations:** Institute of Medicinal Biotechnology, Chinese Academy of Medical Science & Peking Union Medical College, Beijing 100050, China; niuxia@imb.pumc.edu.cn (X.N.); wangxiaomei@imb.pumc.edu.cn (X.W.); niubingyu@imb.pumc.edu.cn (B.N.); wangyucheng@imb.pumc.edu.cn (Y.W.)

**Keywords:** IMB16-4 nanoparticles, poor aqueous solubility, oral bioavailability, anti-liver fibrosis

## Abstract

Liver fibrosis is challenging to treat because of the lack of effective agents worldwide. Recently, we have developed a novel compound, N-(3,4,5-trichlorophenyl)-2(3-nitrobenzenesulfonamido) benzamide referred to as IMB16-4. However, its poor aqueous solubility and poor oral bioavailability obstruct the drug discovery programs. To increase the dissolution, improve the oral bioavailability and enhance the antifibrotic activity of IMB16-4, PVPK30 was selected to establish the IMB16-4 nanoparticles. Drug release behavior, oral bioavailability, and anti-hepatic fibrosis effects of IMB16-4 nanoparticles were evaluated. The results showed that IMB16-4 nanoparticles greatly increased the dissolution rate of IMB16-4. The oral bioavailability of IMB16-4 nanoparticles was improved 26-fold compared with that of pure IMB16-4. In bile duct ligation rats, IMB16-4 nanoparticles significantly repressed hepatic fibrogenesis and improved the liver function. These findings indicate that IMB16-4 nanoparticles will provide information to expand a novel anti-hepatic fibrosis agent.

## 1. Introduction

Liver fibrosis is a common protective response to chronic liver diseases and represents a significant world public health problem [1,2,3,4,5]. It is characterized by excessive production of extracellular matrix (ECM) by hepatic stellate cells (HSCs), which results in cirrhosis and possible death. Currently, liver transplantation is considered the only treatment for end-stage cirrhosis [6]. At present, enormous efforts have been devoted to understanding the underlying molecular mechanisms and developing new therapies of liver fibrosis [7,8,9,10,11,12,13,14,15,16]. So far, no specific medical therapy is approved [17]. Therefore, the search for effective drugs for the treatment of liver fibrosis is very important.

Recently, a novel compound, N-(3,4,5-trichlorophenyl)-2(3-nitrobenzenesulfonamido) benzamide, abbreviated as IMB16-4, was synthesized by our research team. Our study firstly confirmed the anti-fibrotic effect of IMB16-4 on the bile duct ligation (BDL)-induced liver fibrosis rats. However, IMB16-4 displays very poor aqueous solubility and conventional therapy is not effective for the treatment of liver diseases. It has also been reported that drug nanoparticles of <100 nm have novel physical properties and show improvement in permeation through various biological barriers [18]. Therefore, it is necessary to select suitable stabilizers for obtaining stable nanoparticles during the preparation. The pharmaceutical excipients are often used as stabilizers, such as hydroxypropyl methylcellulose (HPMC) [19], hydroxypropylcellulose (HPC) [20], polyvinyl pyrrolidone (PVPK30) [21], polysorbate (Tween 80) [22], and sodium dodecyl sulfate (SDS) [23,24]. Among the strategies used to obtain the nanoparticles, the anti-solvent precipitation method has been explored, and it is easily commercialized with a relatively cheap cost to scale up production [25,26,27,28,29].

Herein, we established IMB16-4 nanoparticles and discussed their therapeutic efficacy on anti-liver fibrosis in rats. PVPK30 were selected as stabilizers to improve the stability, enhance the bioavailability, and increase anti-fibrotic effect. The molecular state and release behavior of IMB16-4 in nanoparticles were systematically studied by transmission electron microscopy (TEM), Fourier transform infrared spectroscopy (FTIR), differential scanning calorimetry (DSC), and X-ray diffraction (XRD). In vivo pharmacokinetic studies in rats were conducted to confirm the increase in oral bioavailability. The anti-fibrotic effect and the underlying molecular mechanisms of IMB16-4 were investigated in rats by testing liver histology, hepatic functions, and the level of fibrotic mediators, including α-smooth muscle actin (α-SMA) and matrix metalloproteinase-2 (MMP-2). We expect that IMB16-4 nanoparticles in this study will help expand a novel anti-hepatic fibrosis agent in the pharmaceutical field.

## 2. Results and Discussion

### 2.1. The Morphology and the Storage Stability of IMB16-4 Nanoparticles

**Figure 1:**
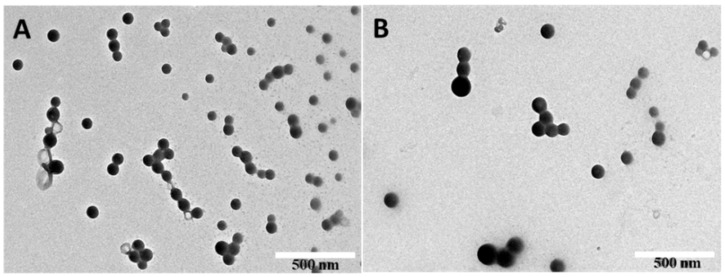
The particle size and morphology of NS. NS (**A**) and NS after one year (**B**) after redispersion by water.

IMB16-4 is soluble in DMF and nearly insoluble in water [30]. Anti-solvent precipitation method was selected to obtain a narrow size distribution particle size with good operability and suitable for large-scale industrial production. PVPK30 was regarded as protective agent to prevent aggregation. PVPK30 was soluble in water and IMB16-4 nanoparticles were difficult to store in solution for a long time. Particle size of IMB16-4 suspension was stabilized within 6 h at 25 °C, and particle size increased over time under shaker condition at 37 °C (Table 1). It indicated that particle size might be growing larger in the digestive tract. IMB16-4 nanoparticles were freeze-dried for long-term preservation, and they can be restored to the original shape after being watered. After freeze-drying, IMB16-4 nanoparticles were called NS for short. As shown in Figure 1, NS displayed a nearly spherical shape with the size of ∼80 nm and excellent size distribution after redispersion (PDI < 0.3) (Table 2). After storing for one year, particle size and size distribution were not obvious changed.

### 2.2. Characterization of IMB16-4 Nanoparticles

**Figure 2:**
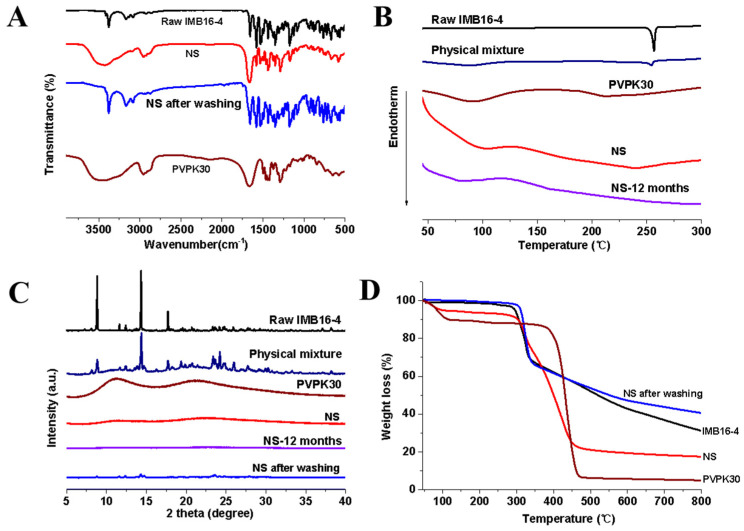
Characterization of pure IMB16-4 and NS: (**A**) FTIR spectra of NS, PVPK30, and pure IMB16-4. (**B**) DSC thermogram of PVPK30, pure IMB16-4, physical mixture, and NS. (**C**) XRD patterns of PVPK30, pure IMB16-4, physical mixture, and NS. (**D**) TGA spectra of NS, PVPK30, and pure IMB16-4.

It is well known that FTIR could provide surface information about materials for identification of chemical groups. Therefore, FTIR can be used to detect structural changes that would lead to changes in bonding between chemical groups [31]. Figure 2A reveals that pure IMB16-4 existed C–H (unsaturated) stretching vibration at 3080~3200 cm^−^^1^ and NH stretching mode at 3375 cm^−^^1^, which were substituted by the C–H aliphatic stretching of PVPK30 at 2950 cm^−^^1^ in NS. Moreover, NH stretching mode at 3375 cm^−^^1^ disappeared in NS. The bands of NS after washing-off PVPK30 reappeared at 3080~3200 cm^−^^1^ and 3375 cm^−^^1^. These changes indicated that IMB16-4 of NS showed no change in chemical groups compared with the pure IMB16-4.

The result of DSC can be seen in Figure 2B. In the case of pure IMB16-4, a single sharp endothermic melting peak at 253~258 °C was observed. The physical mixture of IMB16-4 and PVPK30 showed a slightly narrower melting peak compared with that of pure IMB16-4. PVPK30 showed a broad endothermic peak ranging from 75 to 120 °C due to the presence of residual moisture. However, no trace of an endothermic peak was observed for freeze-dried NS in the DSC curves. After storing at room temperature for a year, no significant changes were seen in the DSC curves of NS.

The XRD method was found to be quite valuable tool for the confirming the crystalline form [32]. As shown in Figure 2C, the diffraction pattern of pure IMB16-4 was highly crystalline as indicated by the numerous peaks. For the physical mixture, the numerous peaks were absolutely attributed to those of pure IMB16-4, confirming that PVPK30 had no effect on the drug crystalline transition. However, no crystalline IMB16-4 was detected in NS. IMB16-4 existed as amorphous in NS. Moreover, to examine the stability of NS, they were stored at room temperature for one year. No significant changes were seen in the XRD pattern and DSC curve, indicating that NS inhibited aggregation and recrystallization for a relatively long time. However, the slight peaks of NS were observed after washing out the PVPK30, indicating the amorphous form of IMB16-4 was difficult to preserve without the stabilizer.

### 2.3. Estimation of IMB16-4 Content in the Nanoparticles by HPLC Analysis

**Figure 3:**
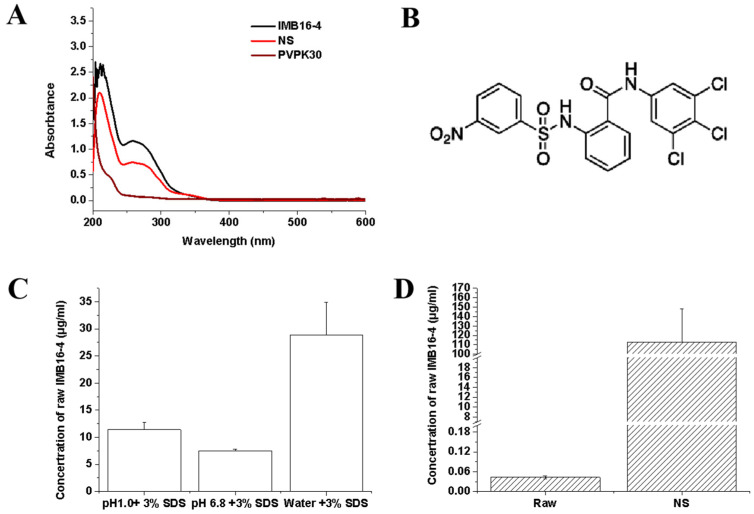
Characterization of pure IMB16-4 and NS: (**A**) UV–Vis absorption spectra of NS, PVPK30, and pure IMB16-4. (**B**) The chemical structural formula of IMB16-4. (**C**) Equilibrium concentrations of pure IMB16-4 and NS in different media containing 3% SDS. (**D**) The equilibrium concentrations of NS and pure IMB16-4 in water at 25 °C.

The actual drug content of NS was quantified by TGA and HPLC analysis. The TGA curves for pure IMB16-4, PVPK30, NS, and NS after washing off PVPK30 were all shown in Figure 2D. The drug-loading fractions were calculated from the ratio of the weight loss to the total initial weight between 40 and 800 °C. PVPK30 showed slight weight loss at 100 °C due to the presence of residual moisture, which agreed with the results of DSC. From the TGA patterns, it is clear that the residual rate of IMB16-4, NS, and NS after washing off PVPK30 was 31%, 17.4%, and 42% at 800 °C, respectively. PVPK30 had almost decomposed entirely by 800 °C. So, the residual rate of NS was mainly attributed to the IMB16-4. The drug loading content of NS was calculated to 35% by TGA. Although the TGA measurement is not accurate to quantify the total drug content of the sample, together with the HPLC, TGA is an important method to detect the drug loading. The actual drug loading of NS was quantified by HPLC. Before HPLC analysis, the UV–Vis spectra of PVPK30 and pure IMB16-4 were detected to confirm the maximum absorption wavelength. As shown in Figure 3B, pure IMB16-4 had typical absorption with a peak at 258 nm and PVPK30 did not affect absorption at 258 nm. As shown in Table 1, the values of drug uptake were 34.9%. After stored one year, the values of drug uptake were slightly reduced to 33.8% (Table 2).

### 2.4. The Equilibrium Concentrations

NS were evaluated in order to demonstrate the powerful effect and emphasized the improvement effect of the nanoparticles on the dissolution rate of IMB16-4. The solubility of pure IMB16-4 at 25 °C in the distilled water was 43.2 ± 9.1 ng/mL [30]. As shown in Figure 3D, the solubility of NS was 113 ± 35.3 μg/mL, which strongly confirmed the excellent ability of IMB16-4 nanoparticles to enhance water-solubility. The possible mechanisms of increasing the solubility have been postulated as the reduction in crystallinity, decreasing the particle size, enhancing the dispersion, and increasing water absorption by the hydrophilic carrier. Pure IMB16-4 possesses a very low solubility in water, which is hard to detect in HPLC. So, it was necessary to analyze the equilibrium concentration of pure IMB16-4 at 25 °C by HPLC-MS/MS analysis (Thermo LTQ XL, Waltham, MA, USA). In addition, the saturation solubility in different medium containing 3% SDS was detected. The equilibrium concentrations of pure IMB16-4 in different medium containing 3% SDS at 37 °C were 28.9 ± 6.0 μg/mL (water), 7.5 ± 0.3 μg/mL (pH 6.8), and 11.4 ± 1.3 μg/mL (pH 1.0), respectively (Figure 3C).

### 2.5. In Vitro Dissolution Study of IMB16-4 Nanoparticles

**Figure 4:**
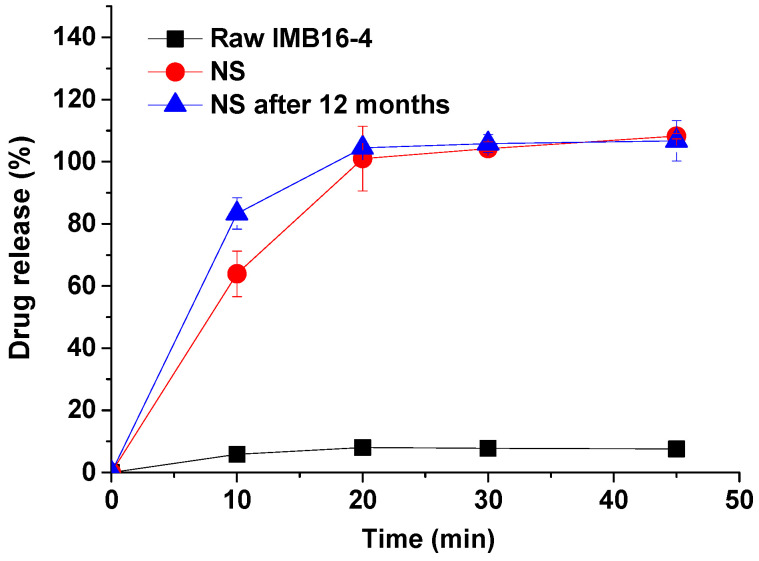
Release profiles of pure IMB16-4 and NS in pH 1.0 containing 3% SDS dissolution medium. Each data point represents the mean ± SD of three determinations.

For nearly insoluble drugs, dissolution can be the rate-controlling step. The dissolution is important to transport the drug particles along the gut and the delivery of pharmaceutical molecules to the epithelium into the blood stream. The equilibrium concentration of pure IMB16-4 was 11.4 ± 1.3 μg/mL in pH 1.0 hydrochloric acid containing 3% SDS (Figure 3C). This satisfied the sink condition when the amount of IMB16-4 was less than 3.42 mg in 900 mL of pH 1.0 hydrochloric acid containing 3% SDS. The first point of pure IMB16-4 in the dissolution test was barely able to be measured by HPLC in the dissolution medium containing less than 3% SDS. In order to compare the dissolution rate and detect the dissolution amount of pure IMB16-4 at each point, the dissolution medium containing 3% SDS was adopted in the study. As shown in Figure 4, the dissolution of NS was very fast in the simulated gastric fluid and almost complete after 20 min. The corresponding dissolution rate of pure IMB16-4 was about 10%. This improved dissolution may be largely attributed to the marked dispersing effect and transforming the crystalline state of IMB16-4 into a noncrystalline state, which is well-known to improve the drug dissolution rate. After storing for one year, the dissolution rate of NS had no obvious change.

### 2.6. Nanoparticles Increase In Vivo Absorption and Liver Accumulation of IMB16-4

**Figure 5:**
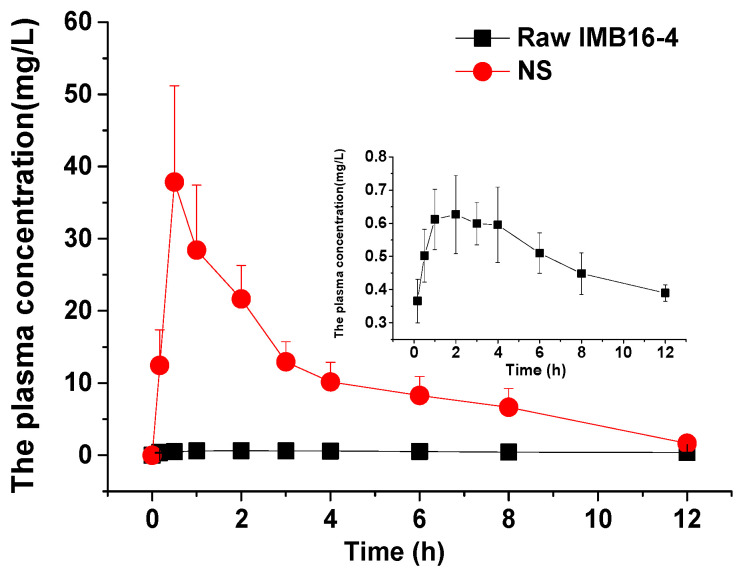
Plasma concentration–time profiles of pure IMB16-4 and NS. Results are expressed as the mean with the bar showing SD values of six rats.

In order to evaluate whether the rapid dissolution of IMB16-4 in vitro can be translated into an increased bioavailability, the in vivo pharmacokinetics of pure IMB16-4 and NS were studied in SD rats. As shown in Figure 5 and Table 3, the AUC_0~12h_ of NS after intragastric administration was increased nearly 26-fold compared with pure IMB16-4, respectively. The Cmax values of NS and pure IMB16-4 were 46.98 ± 13.0 mg/L and 0.68 ± 0.11 mg/L, respectively. Obviously, NS significantly improved the in vivo adsorption of IMB16-4. IMB16-4 nanoparticles greatly increased the oral bioavailability by decreasing the particle size, and increased dispersibility with an increased surface area.

### 2.7. IMB16-4 Nanoparticles Attenuated BDL-Induced Hepatic Fibrosis in Rats

**Figure 6:**
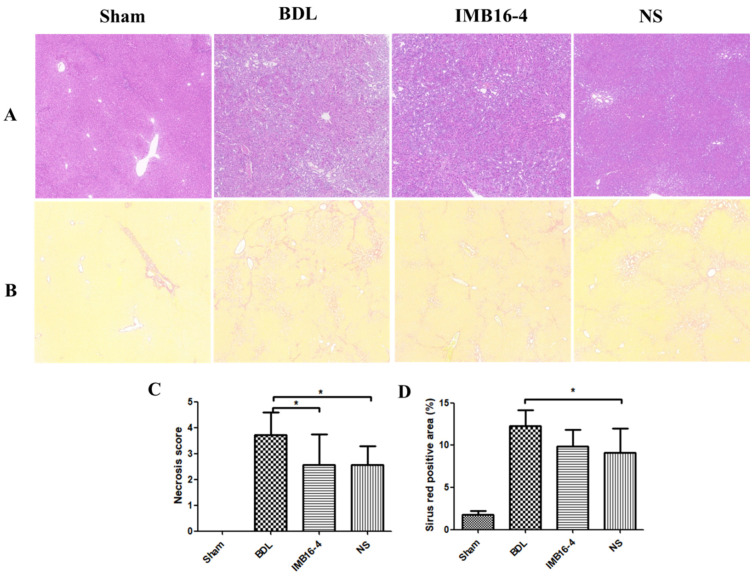
IMB16-4 and NS markedly improved liver fibrosis in BDL rats: (**A**) Liver pathological changes were detected by H&E staining. (**B**) The degree of liver collagen accumulation was determined by Sirius red straining. (**C**) Blinded quantitative assessment hepatic necrosis score. (**D**) The percentage of Sirius red positive areas. * *p* < 0.05 significantly different from BDL group.

The BDL model is widely used to mimic human hepatic fibrosis, which is featured by collagen and extracellular matrix proteins deposition in the liver tissues [33,34]. Liver samples were stained with Sirius red and hematoxylin to evaluate hepatocellular damage and tissue fibrosis. As shown in Figure 6A, rats in the sham group had normal cellular architecture (Ishak score 0). Liver tissue in the BDL group demonstrated cellular damage and centrilobular necrosis. In BDL rats, liver tissue formed occasional bridges in the interstitial spaces, which was marked as Ishak score 3. In some BDL rats, these septa spread to form numerous bridges (Ishak score 4). After being treated with IMB16-4 and NS, liver tissue showed absence of bridging fibrosis; however, interstitial fibrous deposition was slightly observed (Ishak score 2). In a blinded assessment, the BDL-NS group had significantly lower scores for parenchymal necrosis than the BDL group (Figure 6C). In addition, NS attenuated the accumulation of collagen in the liver, which was shown by the substantial decrease in Sirius red positive areas (Figure 6B,D). The results indicate that NS play a therapeutic role in resisting hepatic fibrosis.

### 2.8. IMB16-4 Nanoparticles Improved the Liver Function

The serum levels of ALT, ALP, and AST are sensitive markers for evaluating the liver function in hepatic diseases. As showed in Table 4, rats after BDL surgery showed significant elevation in ALT, ALP, and AST compared with the sham group, indicating considerable liver functional abnormality. After treatment with IMB16-4 or NS, IMB16-4 significantly decreased the level of AST. Furthermore, NS significantly attenuated the levels of ALT, AST, and ALP. There was obvious evidence that NS improved the liver function of rats suffering from hepatic fibrosis.

### 2.9. Effects of IMB16-4 Nanoparticles on Fibrotic Protein Marker in Liver

**Figure 7:**
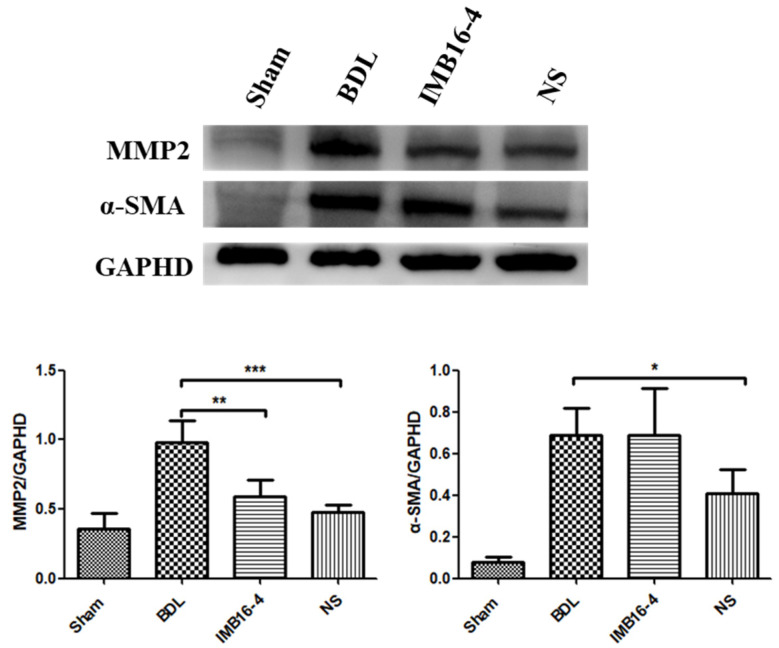
Western blot analysis and semi-quantitation of α-SMA and MMP2 protein levels in liver tissue. The values are expressed as the mean ± SD of four times independent experiments. * *p* < 0.05, ** *p* < 0.01 and *** *p* < 0.001 significantly different from the BDL group.

Activated hepatic stellate cells (HSCs) are considered as key cells responsible for collagen deposition during the process of hepatic fibrogenesis. Protein α-SMA is a marker of HSCs activation. MMP-2 can aggravate the progression of fibrosis via degrading abundant type IV collagen around the HSCs that accelerates HSC activation [35]. As shown in Figure 7, the expression levels of α-SMA and MMP2 in BDL group were significantly increased by BDL operation. However, these increases were prominently suppressed by NS compared with the BDL group. Overall, NS effectively ameliorated the parenchymal necrosis and the excessive collagen though inhibiting HSCs activation. The anti-liver fibrosis effects of NS at lower dose were significantly stronger than those of IMB16-4.

### 2.10. Cytotoxicity of NS on Human Hepatic Stellate Cells (LX-2 cells)

**Figure 8:**
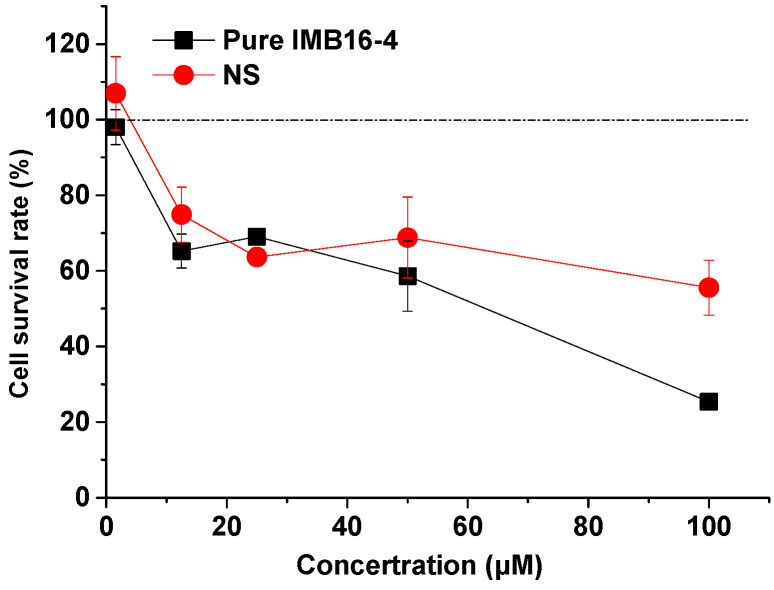
The cell survival rate of LX-2 cells at varying concentrations of pure IMB16-4 and NS. Each data point represents the mean ± SD of three determinations.

Figure 8 shows the survival rate of LX-2 cells after 24 h co-culture. When the concentration of NS and raw IMB16-4 increased, the cell survival was reduced, and this toxic effect observed at high concentrations was absolutely attributed to IMB16-4. The result indicated that NS did not increase toxicities to LX-2 cells.

## 3. Materials and Methods

### 3.1. Materials

IMB16-4 was synthesized by our team (Lot No: IMB20191213, Beijing, China). PVPK30 was purchased from Shanghai Chineway pharma (Shanghai, China). Antibodies for MMP2, α-SMA, glyceraldehyde-3-phosphate dehydrogenase (GAPDH), and HRP-conjugated secondary antibodies against mouse or rabbit IgG were obtained from Proteintech (Wuhan, China). Methanol was chromatographic grade. All other chemicals were the class of reagent grade without further purification. Deionized water was used in all experiments.

### 3.2. Preparation of IMB16-4 Nanoparticles

*N*-(3,4,5-trichlorophenyl)-2(3-nitrobenzenesulfonamido) benzamide (IMB16-4). White solid, m.p.: 256.3~258.1 °C. 1H-NMRδ 10.55 (s, 1H), 10.34 (s, 1H), 8.44 (s, 1H), 8.35 (dd, J = 8.5, 2.1 Hz, 1H), 8.10 (d, J = 7.7 Hz, 1H), 7.88 (s, 2H), 7.78 (t, J = 8.0 Hz, 1H), 7.63 (d, J = 7.6 Hz, 1H), 7.56 (t, J = 7.7 Hz, 1H), 7.43~7.32 (m, 2H). MS *m*/*z*: calcd for C_19_H_12_Cl_3_O_5_S 499.2 [M−H]^−^.

IMB16-4 nanoparticles were produced by anti-solvent precipitation method. Briefly, 1.0 g IMB16-4 was dissolved in 50 mL *N*,*N*-dimethylformamide (DMF). Then, the resulting IMB16-4 solution was dropped into 1000 mL 0.1% PVPK30 solution. Then the mixed solution was kept at room temperature under stirring by using a thermostat magnetic stirrer. After 30 min, the suspension was collected by centrifugation at 12,000 rpm for 30 min to remove DMF. Then, the precipitation was reconstituted by 0.1% PVPK30 solution. Afterwards, the samples were obtained by freeze-drying and used for subsequent tests. The resulting sample was called NS for short.

### 3.3. Long Term Stability Test of IMB16-4 Nanoparticles

The practical size of the NS suspension before freeze-drying was measured at different time periods at 25 °C and 37 °C, respectively.

NS after freeze-drying was kept in desiccators at room temperature (25 ± 2 °C) for 12 months and then evaluated.

### 3.4. Physicochemical Characterization of IMB16-4 Nanoparticles

The size, polydispersity index, and zeta potential of NS were characterized by dynamic light scattering method with Zetasizer Nano ZS instrument (Malvern Instruments, Malvern, UK) at 25 °C.

The morphology of NS was measured by SEM (SU8020, HITACHI, Tokyo, Japan) and TEM (JEM 1200EX, JEOL, Tokyo, Japan). The samples were diluted and poured on copper grids after sonication. The film on the grid was subsequently stained using phosphotungstic acid and allowed to dry at room temperature before TEM examination.

FTIR spectra of IMB16-4 nanoparticles and pure IMB16-4 were collected on FTIR spectrometer (Nicolet IS10, Madison, WI, USA). The IR spectra were obtained over the spectral region 400 to 4000 cm^−1^ in absorbance mode.

DSC analysis of IMB16-4 nanoparticles was carried out with a DSC 1 instrument (Mettler Toledo, Greenville, SC, USA) with a heating rate of 10 °C/min under a nitrogen purge of 50 mL/min. XRD patterns of the samples were collected using an X-ray diffractometer (Brucker D8 Advance, FKB, German) equipped with a CuKα target. Data were obtained from 5° to 40° (diffraction angle 2θ) at a step size of 0.02° and a scanning speed of 4°/min radiation.

To determine the maximum absorption of IMB16-4, UV–Vis absorption spectra were collected with a Hitachi U-3100 spectrophotometer (Hitachi Co. Ltd., Tokyo, Japan). IMB16-4 nanoparticles was analyzed by HPLC (LC-2030, Shimadzu, Japan and was carried out on a Shim-pack C18 column (50 × 2.1 mm, 2 μm) at 25 °C. The mobile phase was consisted of an 80:20 (*v*/*v*) mixture of methanol and 0.1% (*v*/*v*) phosphoric acid solution, the flow rate was 0.3 mL/min and the detection wavelength was 258 nm. The injection volume was 5 μL. The samples were filtered using a 0.22 μm membrane filter before running HPLC analysis.

### 3.5. Drug Loading Analysis by HPLC and TGA

TGA was performed using a TGA/DSC1 instrument (Mettler Toledo, Greenville, SC, USA) under a nitrogen purge of 50 mL/min.

The actual drug loading of NS was also determined by extracting an accurately weighed amount of IMB16-4 nanoparticles with methanol, followed by filtration of the samples and analyzed by HPLC. The standard curve for IMB16-4 was the linear (R^2^ > 0.9999) over the concentration range of 0.2~50.0 μg/mL. The drug loading was calculated according to equation:

Drug loading (%) = (The weight of IMB16-4 in IMB16-4 nanoparticles/the weight of IMB16-4 nanoparticles) × 100.

### 3.6. Equilibrium Concentration Determination

The equilibrium concentration of pure IMB16-4 was measured in triplicate by adding excess amount of IMB16-4 into 5 mL of 3% SDS, 5 mL of pH 1.0 hydrochloric acid containing 3% (*w*/*v*) sodium dodecyl sulfate (SDS), and pH 6.8 phosphate buffer containing 3% SDS at 37 ± 2 °C, respectively. The solutions at equilibrium time (about 72 h) were withdrawn and filtered using a 0.22 μm membrane.

Then, 10 mg of pure IMB16-4 and NS (equivalent to 10 mg IMB16-4) were added into 5 mL of distilled water at 25 ± 2 °C, separately. After 6 h, the solutions were withdrawn and filtered using a 0.22 μm membrane. Then, the solutions were centrifuged at a rate of 15,000 rpm and the supernatants were analyzed by HPLC (LC-2030, Shimadzu, Tokyo, Japan) at 258 nm. IMB16-4 solution in water was diluted with internal standard (4′-Chloroacetanilide) acetonitrile solution and the mixture was vortex-mixed for 10 s and analyzed by HPLC-MS/MS analysis (Thermo LTQ XL, Waltham, MA, USA).

### 3.7. In Vitro Release

The release characteristics were studied by using a USP II paddle method with a dissolution apparatus (ZRS-8LD, Tianda Tianfa Technology Co., Ltd., Tianjin, China). NS sample equivalent to 3 mg IMB16-4 was exposed to 900 mL of pH 1.0 hydrochloric acid containing 3% SDS. The release tests were conducted for 45 min at a constant temperature (37 °C) with a paddle speed of 100 ± 1 rpm in triplicate. Then, 5 mL aliquots were withdrawn and filtered through a 0.22 μm membrane filter and an equal amount of fresh dissolution medium was added to maintain a constant dissolution volume. The withdrawn samples were filtered and analyzed by HPLC at 258 nm.

### 3.8. Pharmacokinetics Study

Sprague-Dawley (SD) rats were supplied by SPF Biotechnology Co., Ltd. (Beijing, China). All animal experiments were performed in accordance with Institutional Review Board for Laboratory Animal Care (Approval No. IMB-20200417D802). SD rats (body weight 200 ± 20 g) were fasted overnight and divided into three groups with 6 rats in each group. Pure IMB16-4 and NS were given at a dose of 100 mg/kg (calculated by IMB16-4), respectively. Before administration, pure IMB16-4 was dispersed in 0.3% sodium carboxyl methyl cellulose aqueous solution. NS was dispersed in distilled water. Then, samples were immediately given orally by gavage. Blood samples were collected from the eye socket vein at time points of 0.17, 0.5, 1, 2, 3, 4, 6, 8, and 12 h after dosing. The plasma samples were collected by centrifuging at 3000 rpm for 10 min. Plasma samples (30 μL) were mixed with 90 μL internal standard (4′-Chloroacetanilide) acetonitrile solution. Then, the mixture was vortex-mixed for 10 s. After centrifugation at 14,000 rpm for 10 min, 5 μL of the supernatant was transferred into 95 μL of acetonitrile. After the vortex, 1 μL of mixed liquor was subjected to HPLC-MS/MS analyzer (Thermo ITQ XL, Waltham, MA, USA). The mobile phase was comprised of acetonitrile and 10 mM ammonium acetate aqueous solution in gradient elution. The column temperature was 30 °C and the flow rate was 0.3 mL/min. The standard curve for IMB16-4 was the linear (R^2^ > 0.992) over the concentration range of 0.3~1200.0 ng/mL. The quantitative ion pair of IMB16-4 and internal standard quantitative were *m*/*z* = 499.9/196.0 and *m*/*z* = 168.04/126.1, respectively. The pharmacokinetic parameters were obtained using statistic software DAS2.0.

### 3.9. Pharmacodynamics Study

#### 3.9.1. Bile Duct Ligation (BDL) Surgery in Rats

SD rats (180 ± 20 g) were supplied by HFK Biotechnology Co., Ltd. (Beijing, China). All animal experiments were performed in accordance with Institutional Review Board for Laboratory Animal Care (Approval No. IMB-2020111306D6).The rats were maintained under a specific pathogen-free (SPF) environment with a 12 h light/dark cycle. Hepatic fibrosis model was established by bile duct ligation and performed under anesthesia with isoflurane [36]. Twenty-eight SD rats and were divided into four groups including the sham group (*n* = 7), BDL group (*n* = 7), BDL- IMB16-4 group (*n* = 7), and BDL-NS group (*n* = 7). Rats in the sham group received laparotomy but without BDL. The BDL-IMB16-4 group received daily gavages with 600 mg/kg/day IMB16-4 in 0.3% sodium carboxymethyldellulose solution. The BDL-NS group received NS by feeding the animal with pellet food containing 15 mg/kg/day of IMB16-4. After 14 days, blood samples were collected from the abdominal aorta, and liver tissue was also collected from all groups. All samples were stored at −80 °C for further analysis.

#### 3.9.2. Serum Biochemical Parameters

To evaluate the liver function, the alanine aminotransferase (ALT), aspartate transaminase (AST), and alkaline phosphatase (ALP) were measured with kits (Zhongsheng Beikong Biotechnology, Beijing, China).

#### 3.9.3. Histological Analysis of Liver Tissue

Liver tissue was fixed in formalin, dehydrated, and embedded in paraffin. Then, liver sections were stained with hematoxylin and eosin (H&E) and Sirius red, respectively. The degrees of fibrosis were evaluated in a blinded manner. The sections stained with Sirius red were observed at low magnification and calculate the percentage of hepatic fibrous area using image J free software.

#### 3.9.4. Western Blot Analysis

Liver tissue samples were lysed using RIPA lysate buffer containing protease inhibitor on ice and then centrifuged (15,000 rpm) at 4 °C for 20 min. The supernatant was collected and protein concentration was determined with a BCA protein quantitation kit (Beyotime, Shanghai, China). Samples containing at least 30 μg total proteins were applied to 10% SDS-PAGE gel. Then, the protein bands were transferred to a polyvinylidene difluoride (PVDF) membrane (Millipore Corp., Atlanta, GA, USA). After blocking with 5% skim milk for 1 h, the membranes were incubated with the desired primary antibodies overnight at 4 °C. Peroxidase (HRP)-conjugated secondary antibodies was incubated for 1 h at room temperature. Finally, the protein bands were visualized with an ECL Western blot substrate kit. The pictures were observed on a chemiluminescence imaging system (Tanton, Beijing, China). Image J free software was used to analyze the protein bands densities.

### 3.10. Cytotoxicity Assays

LX-2 cells were chosen for in vitro cytotoxicity testing of raw IMB16-4 and NS. LX-2 cells were cultured in Dulbecco’s Modified Eagle’s Medium (DMEM, high glucose, 10% fetal bovine serum) and incubated at 37 °C in an atmosphere of 5% CO_2_, and the medium was changed every 2 days. In each test, the cell suspension was diluted with DMEM, seeded into 96-well plates at 100 μL per well, and incubated for 24 h. Then, NS and IMB16-4 suspensions containing different concentrations were added to 96-well plates at 100 μL per well and incubated for 24 h. Then, 10 μL CCK8 solution was added to 96-well plates and incubated for 2 h. Finally, the absorbance was determined at 450 nm (*n* = 3), and the cell survival was calculated according to equation. An optical microscope (BioTek, SYNERGYH1, Vermont, VT, USA) was used to examine the cell survival.

Cell Survival (%) = Absorbance of sample/Absorbance of control × 100

### 3.11. Statistical Analysis

All quantitative data are presented as mean ± SD with a minimum of three independent samples and analyzed by one-way analysis of variance (ANOVA). *p* values of < 0.05 were regarded as statistically significant. Statistical analysis was performed in GraphPad Prism 5.

## 4. Conclusions

In summary, we successfully developed IMB16-4 nanoparticles to ameliorate anti-hepatic fibrosis using a simple process. IMB16-4 nanoparticles were stably preserved for a long time. The experimental results suggest that NS exhibited rapid diffusion into the dissolution medium, greatly improved the bioavailability, and increased the anti-hepatic fibrosis effect. The exploration of NS in this paper provides a foundation for further development on the effective dose, oral administration, and administration frequency of IMB16-4 nanoparticles to resist liver fibrosis.

## Figures and Tables

**Table 1:** The stability of NS suspension at different temperatures.

Time (h)	25 °C		37 °C	
	Mean PS ± SD	PDI	Mean PS ± SD	PDI
0	112.4 ± 43.9	0.083	124.8 ± 40.0	0.073
2	118.0 ± 38.5	0.078	119.2 ± 41.2	0.078
4	119.8 ± 40.5	0.093	301.8 ± 61.8	0.373
6	117.7 ± 47.8	0.108	268.2 ± 172.7	0.606

**Table 2:** Particle size (PS), zeta potential measurement (ZP), and the content of NS after freeze-drying.

Samples	Mean PS ± SD	PDI	Mean ZP ± SD	Degree of Drug Loading (%)
NS	164.3 ± 78.5	0.150	−15.6 ± 6.7	34.9
NS after one year	174.3 ± 83.6	0.218	−13.6 ± 5.8	33.8

**Table 3:** Pharmacokinetic parameters of pure IMB16-4 and NS after oral administration, mean ± SD (*n* = 6).

Pharmacokinetic Parameter	Pure IMB16-4	NS
C_max_ (mg/L)	0.68 ± 0.11	46.98 ± 13.0
T_max_ (h)	2.25 ± 1.33	1.17 ± 1.08
AUC_0~12h_ (mg/L h)	5.95 ± 0.57	155.5 ± 70.5

**Table 4:** Serum biochemical markers of bile duct ligated (BDL)-treated rats, Mean ± SD, *n* = 7.

	Sham	BDL	BDL-IMB16-4	BDL-NS
ALT (U L^−1^)	39.71 ± 9.25	80.29 ± 12.80 ^###^	78.86 ± 12.93	56.00 ± 15.46 *
AST (U L^−1^)	128.86 ± 49.81	405.00 ± 57.07 ^###^	317.43 ± 74.65 *	297.71 ± 95.08 *
ALP (U L^−1^)	191.14 ± 38.10	311.57 ± 65.91 ^###^	328.00 ± 47.62	235.00 ± 36.97 *

^###^*p* < 0.001, significantly different from the sham group. * *p* < 0.05, significantly different from the BDL group.

## Data Availability

Data is contained within the article.
